# Towards sub-millisecond cryo-EM grid preparation

**DOI:** 10.1039/d2fd00079b

**Published:** 2022-08-03

**Authors:** David P. Klebl, Robert W. Kay, Frank Sobott, Nikil Kapur, Stephen P. Muench

**Affiliations:** a School of Biomedical Sciences, Faculty of Biological Sciences & Astbury Centre for Structural and Molecular Biology, University of Leeds UK S.P.Muench@leeds.ac.uk; b School of Mechanical Engineering, University of Leeds Leeds UK; c School of Molecular and Cellular Biology, Faculty of Biological Sciences & Astbury Centre for Structural and Molecular Biology, University of Leeds UK

## Abstract

Sample preparation is still a significant problem for many single particle cryo-EM workflows and our understanding and developments in the area lag behind that of image processing and microscope design. Over the last few years there has been growing evidence that many of the problems which occur during sample preparation are during the time the sample resides within the thin film created during the conventional blotting process. In parallel, faster grid preparation approaches have been developed for time-resolved cryo-EM experiments allowing for non-equilibrium intermediates to be captured on the ms timescale. Therefore, an important question is how fast can we prepare suitable grids for imaging by cryo-EM and how much does this mitigate the problems observed in sample preparation? Here we use a novel approach which has been developed for time-resolved studies to produce grids on an estimated sub-1 ms timescale. While the method comes with its own challenges, a 3.8 Å reconstruction of apoferritin prepared with the ultrafast method shows that good resolutions can be achieved. Although several orders of magnitude faster than conventional approaches we show using a ribosome sample, that interactions with the air–water interface cannot be avoided with preferred orientations still present. Therefore, the work shows that faster reactions can be captured but poses the question whether speed is the answer to problems with sample preparation.

## Abstract 2

First results from a new cryo-EM grid preparation method are presented, significantly faster than previous methods. The short time delay has the potential to improve grid preparation for challenging samples and to allow faster time-resolved EM.

## Graphical abstract

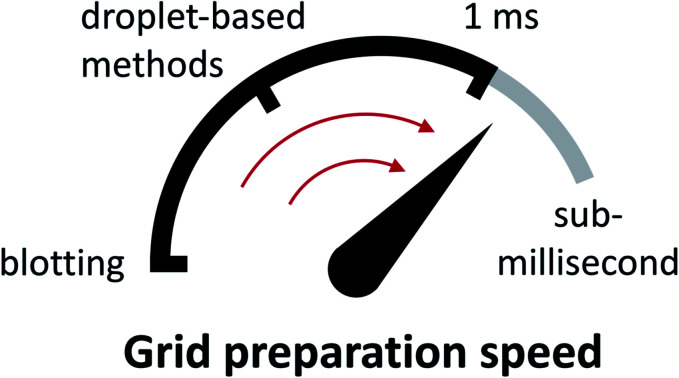

## Introduction

The developments in single particle cryo-EM data processing, camera technology and microscope design have made sub-4 Å structures more accessible and allowed for sub-2 Å for well-behaved protein samples. Although it is clear that the traditional sample preparation approach works for some proteins, this is not the case for all and many cryo-EM workflows stall at the grid preparation stage where problems can occur with sample degradation, preferred orientation and poor distribution within the ice. In conventional cryo-EM grid preparation by blotting, the sample is applied to the grid manually, excess liquid is blotted away with filter paper and the thin film of liquid is then vitrified by plunging in liquid ethane.^1^ While this method has succeeded as a simple way to generate the thin liquid films needed for cryo-EM, the process is slow and typically takes around 10 s. As a result, the sample is exposed to the air–water interface (AWI) or to the support film for an extended amount of time, during which the majority of samples interact with the AWI.^2^ These interactions can lead to many of the problems seen such as preferred orientation and protein denaturation or modification, potentially with detrimental impact on structure determination.^3^ The blotting process can also concentrate the protein on the grid in a variable way meaning experiments can vary wildly in terms of protein concentration used and this can further hinder and slow grid optimisation.^4^

To remove some of the issues with blotting other approaches have been developed, for example the Vitrojet which uses a pin printing approach.^5^ This has also reduced the speed of sample preparation into the low seconds timeframe. Faster grid-preparation methods have been developed, for example with the Spotiton system which has been commercialised as the Chameleon.^6^ The fastest approaches are droplet-based methods, where the sample is atomised and applied to the grid as spray droplets, achieving delay times on the order of 10 ms between sample application and vitrification.^7^ Such fast grid preparation has been shown to alleviate some of the problems associated with the AWI, like denaturation and preferred orientation.^8^ However, interactions between the sample and the AWI cannot be eliminated even with the fastest methods currently available.^9^ Diffusion within the thin liquid layer prior to vitrification is fast, so particles will have multiple opportunities to interact with the AWI even on a timescale of 1 ms.^10^ Only an approach that is significantly faster than currently available methods would have the potential to outrun the interactions with the AWI. Sub-millisecond grid preparation could give insight whether the detrimental influence of the AWI can be avoided entirely based on speed.

There has also been a drive to develop grid making approaches for time-resolved cryo-EM, where short delays between sample application and vitrification are needed to trap short-lived intermediates, typically in the 10–1000 ms timeframe.^11,12^ However, time-resolved studies would benefit from even faster cryo-EM grid preparation, especially for systems that rely on rapid mixing for reaction initiation. Many protein–ligand systems fall in this category, where initial ligand binding is rapid and the protein structure subsequently changes (induced fit).^13^ Current methods for time-resolved EM, while capable of trapping biochemical states, usually do not have the necessary short dead time (sub-millisecond) to analyse faster and more subtle conformational rearrangements.

Here, we report preliminary results from ultrafast grid preparation. We confirm the shorter delay time between mixing (or sample application) and vitrification using a model reaction of actomyosin with high ATP concentrations. The estimated dead time is in the sub-millisecond range and we demonstrate that high-resolution reconstructions are possible by resolving apoferritin to 3.8 Å. This new approach has allowed the first sub ms analysis of interactions between particles and the AWI based on particle orientations of a bacterial ribosome sample. We show that although we can now produce grids orders of magnitude faster than traditional approaches which is beneficial for time-resolved EM, we cannot fully out-run the AWI. However, given the other benefits that have been reported for rapid grid preparation this is the first step in being able to better parameterise and study the many processes that occur during sample preparation.

## Materials and methods

### Kinetic modelling of ATP-induced actomyosin dissociation

**Fig. 1:**
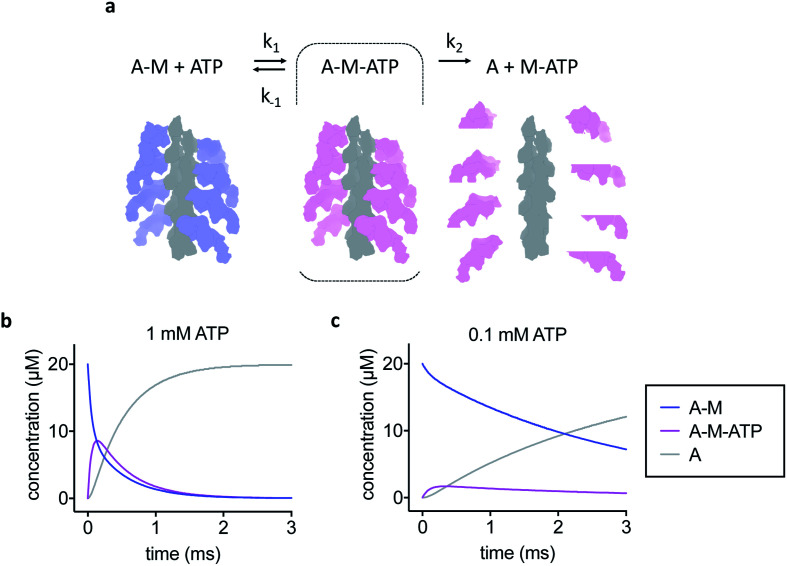
Kinetic model of actomyosin dissociation. (a) Schematic and kinetic model. A-M = actomyosin, A-M-ATP = actomyosin–ATP complex, A = actin, M-ATP = myosin–ATP complex. F-actin is shown in grey, nucleotide free myosin shown in blue, ATP-bound myosin shown in pink. The A-M-ATP state is highlighted with a dashed line box. (b) Kinetic modelling of the reaction for 1 mM initial ATP concentration or (c) 0.1 mM initial ATP concentration. Modelling shows that high ATP concentration is needed to enrich the A-M-ATP state of interest, but also results in a faster overall reaction.

The simple kinetic model used is shown in Fig. 1a. We assumed fast binding of ATP to actomyosin, with a second order rate of *k*_1_ = 10^7^ M^−1^ s^−1^.^14^ The equilibrium constant of ATP-binding has been estimated as *K*_1_ = 500–2000 M^−1^.^15,16^ Based on this, we assume a rate of *k*_−1_ = 6000 s^−1^ for ATP dissociation from the actomyosin complex. Finally, the maximum rate of ATP-induced dissociation of skeletal actomyosin has been reported as >2000 s^−1^, we used *k*_2_ = 3500 s^−1^ in this work and treated the dissociation reaction as irreversible.^16^ Note that the rate constants here are only estimates, the true values may differ and also depend on temperature and buffer conditions, but we believe that the approximations are appropriate for this work.

### Sample preparation

Monomeric rabbit G-actin was obtained as described previously.^17^ G-actin was polymerised to F-actin as described previously.^18^ Rabbit skeletal myosin S1 (A1 fraction) was prepared as described previously.^19^ The actomyosin complex was obtained by mixing F-actin and myosin S1 in a 1 : 1 molar ratio at final concentrations of 40 μM, all dilutions were made in 10 mM MOPS, 2 mM MgCl_2_, 0.1 mM EGTA, 50 mM potassium acetate pH 7.

Disodium ATP was prepared as a 100 mM stock solution in water at pH 7, stored at −20 °C and diluted in 10 mM MOPS, 2 mM MgCl_2_, 0.1 mM EGTA, 50 mM potassium acetate pH 7 to the desired concentration (2 or 5 mM) before use. In the mixing experiments, 40 μM actomyosin was mixed with 2 mM ATP or with 5 mM ATP/5 mg mL^−1^ horse spleen apoferritin (as a marker for mixing) in a 1 : 1 volume ratio. This gave final concentrations of 20 μM actomyosin and 1 mM ATP or 2.5 mM ATP/2.5 mg mL^−1^ apoferritin, respectively.

Horse spleen apoferritin was purchased from Sigma Aldrich (A3660), and exchanged into 30 mM HEPES, 150 mM NaCl pH 7.5 by ultrafiltration using a spin concentrator (100 kDa molecular weight cut-off). For grid preparation, apoferritin was diluted to 10 mg mL^−1^ in 30 mM HEPES, 150 mM NaCl, 1% (v/v) glycerol, 1 mM DTT pH 7.5.

The *Escherichia coli* ribosome sample was purchased from New England Biolabs (P0763S). For grid preparation, the sample was diluted to 6.6 mg mL^−1^ in 50 mM HEPES, 8 mM MgCl_2_, 100 mM potassium acetate pH 7.5.

### Cryo-EM grid preparation

The method for ultrafast cryo-EM grid preparation is currently under development and will be described elsewhere in detail.

### Cryo-EM data collection and processing

Quantifoil R1.2/1.3 grids were used after glow discharge in a Cressington 208 carbon coater with a glow-discharge unit for 30–90 s at 0.1 mbar and 15 mA, or in a tergeo plasma cleaner with indirect plasma in a nitrogen/oxygen/argon mix for 1 min at 15 W power. All cryo-EM data were collected on a Titan Krios microscope equipped with a Falcon IV detector in counting mode. All data processing was done in RELION 3.1, RELION’s implementation of MotionCor2 was used for correcting micrographs for beam-induced motion and CTFFIND 4.1 was used to estimate the contrast transfer function (CTF).^20–23^ The main data collection and processing parameters are listed in Tables 1–3.

**Table 1:** Cryo-EM data collection and processing parameters for actomyosin dissociation experiments

Data collection and processing	A-M with 1 mM ATP	A-M with 2.5 mM ATP/apoferritin
Magnification	96 000	96 000
Voltage (kV)	300	300
Pixel size (Å)	0.82	0.82
Total fluence (e^−^ Å^−2^)	30	34
Number of fractions	30 (EER)	26 (EER)
Exposure time (s)	4	4
Number of micrographs	199	1416
	307	(320 ‘good’)
	102	
Initial number of segments	19 165	9678
Final number of segments	2682 (A-M/A-M-ATP)	2921 (A-M/A-M-ATP)
	770 (A)	349 (A)
Resolution (FSC = 0.143) (Å)	16 (A-M/A-M-ATP)	—
	24 (A)	

**Table 2:** Cryo-EM data collection and processing parameters for apoferritin

Data collection and processing	Apoferritin
Magnification	96 000
Voltage (kV)	300
Pixel size (Å)	0.82
Total fluence (e^−^ Å^−2^)	40
Number of fractions	43 (EER)
Exposure time (s)	6
Number of micrographs	511
Initial number of particles	45 482
Final number of particles	9672
Resolution (FSC = 0.143) (Å)	3.8

**Table 3:** Cryo-EM data collection and processing parameters for the ribosome sample

Data collection and processing	Ribosome
Magnification	47 000
Voltage (kV)	300
Pixel size (Å)	1.8
Total fluence (e^−^ Å^−2^)	11
Number of fractions	80 (EER)
Exposure time (s)	3
Number of micrographs	110
Initial number of particles	10 303
Final number of particles	1123 (70S)
	2809 (50S)
	1803 (30S)
Resolution (FSC = 0.143) (Å)	15 (70S)
	12 (50S)
	20 (30S)

For the dataset of actomyosin (A-M) with 1 mM ATP, data from 3 different grids were collected and combined for processing. Filaments were manually picked and after one round of helical 2D classification and one round of helical 3D classification, ‘good’ particles were taken forward to generate a consensus 3D reconstruction (using a regularization value of *T* = 2). Non-helical focussed classification with a mask covering the central myosin binding site was then used to distinguish between actomyosin complex (A-M or A-M-ATP) and free actin subunits (A). Finally, helical reconstructions were generated for the A-M/A-M-ATP and A classes (with *T* = 2). The dataset of A-M with 2.5 mM ATP/2.5 mg mL^−1^ apoferritin was processed in the same way, except that only a subset of micrographs with good CTF estimation parameters were used.

For the apoferritin dataset, particles were picked using the general model in crYOLO.^24^ After one round of 2D classification, ‘good’ particles were taken forward to refinement with octahedral symmetry. The final reconstruction was obtained after 3 rounds of Bayesian polishing and CTF refinement. To validate the apoferritin reconstruction, PDB 6rjh was docked into the reconstruction, and the Fourier shell correlation between map and model was determined using the validation tool in phenix.^25^

For the ribosome dataset, particles were picked using the general model in crYOLO. 2D classification revealed different ribosome species, so all picked particles were subjected to 3D classification using a 50S ribosome reconstruction as reference. From this classification, a subset of ‘good’ 50S particles was selected and taken forward to refinement. The other particles were subjected to a second round of 3D classification using a 70S ribosome reconstruction as reference. From this classification, a subset of ‘good’ 70S particles was selected and taken forward to refinement. The remaining particles, after an additional 2 rounds of 3D classification with a 30S ribosome reconstruction as reference, produced a subset of ‘good’ 30S particles which was taken forward to refinement. Cryo-EM reconstructions were visualised using ChimeraX, plots of angular orientation were generated using a script adapted from Naydenova and Russo.^10,26^

## Results

To develop our current work in sample preparation and time-resolved methodologies we developed a new approach for grid preparation where we asked if it was possible to produce grids in a sub-ms timescale. We followed on from our previous work on ATP-induced actomyosin dissociation by time-resolved cryo-EM,^18^ and reasoned that faster time delays than currently possible would be needed to trap an ATP-bound actomyosin complex intermediate. This gave us a system that would be able to approximate the speed in which the grids are prepared and give a proof of principle for time-resolved studies. When actomyosin and ATP are mixed, the myosin motor binds ATP and the affinity for the actin filament is reduced dramatically. As a result, the actomyosin complex dissociates. It has been proposed that two distinct actomyosin-ATP (A-M-ATP) intermediates exist before the complex dissociates, both with short lifetimes.^15^ For simplicity, we only consider one intermediate, and we use rate constant estimates based on biochemical kinetic data to model the reaction (Fig. 1a–c).

Based on the modelling, dead-times substantially shorter than 5 ms will be required to enrich the A-M-ATP intermediate(s) of interest. Note that the overall reaction rate can be slowed by using low ATP concentration, as expected for a bimolecular reaction. We had previously used 100 μM ATP in time-resolved experiments to obtain an approximate pseudo first order of about 150 s^−1^. However, the A-M-ATP intermediate is only populated significantly at high ATP concentration when the overall reaction is fast. For example, at ATP = 1 mM, the reaction has almost completed (or reached a steady state) after 1 ms (Fig. 1b).

**Fig. 2:**
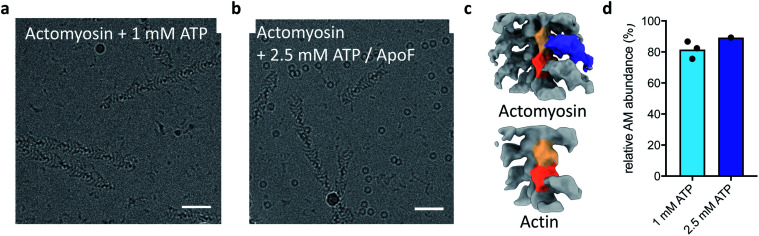
Ultrafast cryo-EM grid preparation captures actomyosin before dissociation. (a) Raw micrograph of actomyosin mixed with 1 mM ATP. (b) Raw micrograph of actomyosin mixed with 2.5 mM ATP and apoferritin as a marker. The scale bars in (a) and (b) correspond to 50 nm. (c) 3D classification result from the 1 mM ATP data, showing clear separation into an actomyosin class (top) and actin class (bottom). (d) Relative actomyosin particle number in the 1 mM ATP and 2.5 mM ATP datasets, showing that only a small fraction of actomyosin complex has dissociated at the time of vitrification.

Next, we devised a grid preparation approach with significantly shorter delay time between sample application and vitrification. We used our novel method for ultrafast grid preparation to mix actomyosin (20 μM final concentration) with a high concentration of ATP (1 mM final concentration). Cryo-EM images revealed a large fraction of actomyosin complex, in the A-M or A-M-ATP state (Fig. 2a). This confirmed that the method was indeed significantly faster than previously described droplet-based methods for plunge freezing.

A limitation of this approach is that the A-M and A-M-ATP states may only be distinguished unambiguously at high resolutions and could appear the same in raw images. To ensure that the two reactants A-M and ATP have mixed on the very short timescale of grid preparation, we prepared grids with apoferritin added to the ATP solution as a marker. The resulting micrographs showed colocalization of A-M complex and apoferritin, indicating that mixing had occurred of the A-M complex with the ATP/apoferritin solution (Fig. 2b). Classification of the data allowed quantification of actomyosin complex and free actin. Despite the low resolution (∼15 Å) of the reconstructions, actomyosin and free actin could be separated well (Fig. 2c). An average of 81% of actin subunits from the 1 mM ATP data had myosin bound (Fig. 2d). A similar fraction of actomyosin complex was found in the 2.5 mM ATP data (Fig. 2d), well within the 95% confidence interval of the 1 mM ATP data.

Comparison with the kinetic model allowed an estimation of the time delay between mixing and vitrification. The observed abundance of actomyosin complex was much higher than the fraction of A-M and A-M-ATP expected from the kinetic model after 1 ms at 1 mM ATP, the model even predicts a lower abundance after 1 ms at 0.1 mM ATP (15% or 74% A-M + A-M-ATP for 1 mM ATP or 0.1 mM ATP, respectively). While this simple estimation of the time delay does not account for other factors, for example cooling of the reaction, it strongly suggests that our approach was working in the sub-ms timescale and demonstrates mixing and freezing on this timescale for the first time in cryo-EM.

**Fig. 3:**
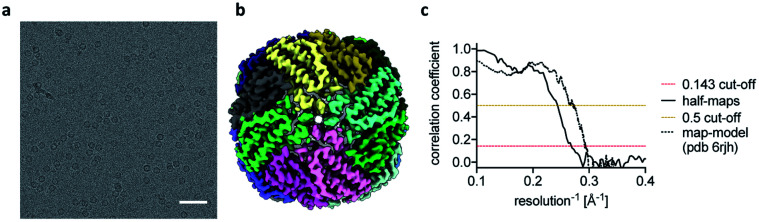
Ultrafast cryo-EM grid preparation of apoferritin. (a) Representative micrograph of apoferritin prepared by ultrafast grid preparation. The scale bar corresponds to 50 nm. (b) Reconstruction of apoferritin at 3.8 Å resolution, coloured by subunit. (c) Fourier shell correlation (FSC) curve for the 3.8 Å apoferritin reconstruction, showing correlation between half-maps as solid black line, and correlation between the final map and PDB 6rjh as dotted black line. The 0.143 FSC threshold is marked as dashed red line, the 0.5 FSC threshold as dashed yellow line.

The reconstructions of the actomyosin complex are at low resolutions, owing to challenges in data collection from ultrafast prepared grids, which will be discussed elsewhere. To demonstrate that the ultrafast grid preparation method is in principle suitable for high-resolution cryo-EM, we prepared and screened grids of apoferritin and collected a small dataset of 511 micrographs. The resulting reconstruction reached a resolution of 3.8 Å (Fig. 3), similar to previous apoferritin structures from fast grid preparation.^27^ These data show that although in the early stages of development it is possible to produce grids in sub-ms time-scale and that they are suitable (although not optimal yet) for structure determination.

**Fig. 4:**
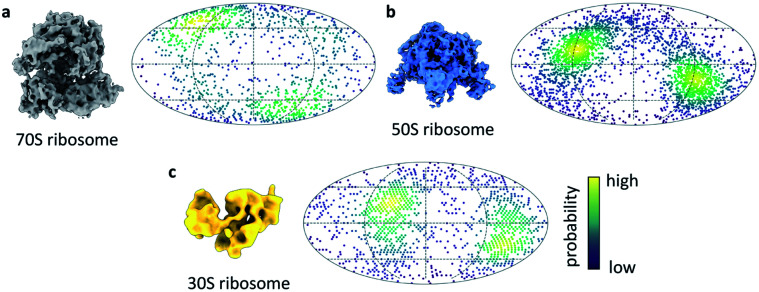
Ribosome orientations from ultrafast grid preparation. Reconstruction and angular orientation in Mollweide projection for (a) 70S, (b) 50S and (c) 30S ribosome particles. The density of angular orientations is indicated by colour, high density (probability) is shown in yellow, low density in dark blue.

We next turned our attention to sample behaviour on the grid, as we know a sub-millisecond timescale has the potential to reduce the interactions between particles and the AWI significantly because these interactions are ultimately limited by diffusion of particles within the thin film. The timescale of diffusion across the thin film has been estimated to occur on a timescale similar to our ultrafast grid preparation approach.^10^ To test if the very short exposure time to the AWI in our new ultrafast grid preparation method could prevent or reduce further interactions with the AWI, we prepared grids of a bacterial ribosome sample. The sample contained a mixture of 70S, 50S and 30S ribosome, as observed previously.^9^ Collection of a small dataset allowed us to determine whether the particles still adopted preferred orientations. In the case of all three ribosome species, there were still clear preferred angular orientations (Fig. 4a–c). While preferred orientation to this extent is unlikely to limit resolution or interfere with 3D reconstruction, it showed that even during the very short residence time in the thin film, the particles did interact with the AWI and were not randomly oriented. We note that the angular distributions are bimodal, probably reminiscent of particles binding to the AWIs on either side of the thin film equally (to a first approximation), and suggesting there is one preferred orientation for each of the ribosome species.

## Conclusions

Sample preparation is still a major limiting factor in single particle cryo-EM and there is currently no general approach for avoiding interactions between the sample and the AWI. To better understand what role the speed of grid preparation plays, and whether very high speeds can eliminate interactions between the sample and the AWI entirely, we developed a new approach which also has the potential of trapping sub-ms non-equilibrium reaction states through rapid mixing and freezing approaches. Here we present the first results from a new cryo-EM grid preparation method, the fastest approach to prepare cryo-EM grids to date. We first studied the actomyosin complex whose kinetics are well characterised and allowed us to estimate the speed at which the grids are prepared. Based on the large fraction of myosin-bound actin subunits after mixing with high concentrations of ATP, we estimate the timescale from mixing to freezing is less than 1 ms. Optimisation of this new grid preparation approach is ongoing, along with further work to determine the speed of the method. We anticipate that the faster speed will expand the scope of time-resolved experiments compared to current droplet-based methods which are limited to the ∼10 ms timescale. This may be particularly valuable for ligand-binding or enzymatic reactions where initial complex formation is fast and highly concentrated ligand solutions are available. Currently, the ultrafast grid preparation method only offers a fixed, short delay time from mixing to freezing. In cases where a reaction is to be followed over different timepoints, a method to vary the delay between mixing and freezing would be desirable.

We next investigated if this approach could produce ice of sufficient quality to permit structure determination at a modest resolution and show we are able to produce a ∼4 Å structure of apoferritin. Therefore, although in development, going sub-ms does not preclude the ability to generate ice of sufficient quality for modest resolution cryo-EM structures (with further optimisation promising improved resolutions). We estimate that the exposure time of the particles to the AWI is similar to the time estimated by the actomyosin dissociation experiment, in the sub-millisecond range. Diffusion of a large particle like the ribosome in a thin film of 50 nm has been estimated to lead to about 10 contacts with the AWI per millisecond.^10^ The observed preferred orientation in the ribosome dataset suggests that the ribosome particles bind the AWI within the first few contacts. However, the equilibration of particle orientation at the AWI has been shown to be highly protein-specific.^9^ For some samples, substantial improvements in particle orientation have been observed on the timescale of 10s to 100s of milliseconds.^8,28^ In these cases of slower AWI binding, we expect sub-millisecond grid preparation to give further improvements.

Although we did not determine ice thickness of the ultrafast prepared grids by tomography, we estimate it between 50 and 200 nm, which allows calculation of the expected number of particles per image based on the molar concentration (Table 4). This demonstrates that to a first approximation, there is neither a large concentration nor depletion of particles on the grid when using the ultrafast grid preparation method. In principle, this could alleviate the need for screening different particle concentrations, as typically done for conventional cryo-EM grid preparation. The discrepancy in the case of actomyosin may be due to the tendency of filaments to form bundles and large aggregates.

**Table 4:** Expected *versus* observed particle number per micrograph. The expected particle number was calculated based on molar concentration, pixel size, the field of view and is linearly dependent on ice thickness. The observed particle number is the number of picked particles divided by the number of micrographs. Expected particle numbers for the ribosome are accounting for dissociation of 66% of ribosome particles

	A-M with 1 mM ATP	A-M with 2.5 mM ATP	Apoferritin	Ribosome
Concentration (μM)	20	20	22.5	4.9 (70S)
Expected particle no. per micrograph (50 nm ice thickness)	68	68	76	133
Expected particle no. per micrograph (200 nm ice thickness)	272	272	306	530
Observed particle no. per micrograph	32	30	89	94

Whether for fast time-resolved cryo-EM, to better understand the interactions between particles and the AWI, or to minimise AWI-induced problems for challenging samples, we believe that the new ultrafast grid preparation method is a valuable addition to the repertoire of cryo-EM sample preparation.

This work constitutes a first proof-of-principle and by optimising the method and making it more robust we hope that it will allow routine high-resolution and high-speed grid preparation, with more random particle orientations and minimal perturbation of the sample by the air–water interface.

## Author contributions

Conceptualization: DPK & SPM. Methodology: RWK & NK. Investigation: DPK. Supervision: RWK, FS, NK & SPM. Writing – original draft: DPK. Writing – review & editing: all authors.

## Conflicts of interest

There are no conflicts to declare.
